# Dual Roles of an Algal Farming Damselfish as a Cultivator and Opportunistic Browser of an Invasive Seaweed

**DOI:** 10.1371/journal.pone.0109007

**Published:** 2014-10-15

**Authors:** Kimberly A. Peyton, Lauren M. Valentino, Karen P. Maruska

**Affiliations:** 1 Hawaii Institute of Marine Biology, University of Hawaii – Manoa, Kaneohe, Hawaii, United States of America; 2 Department of Biological Sciences, Louisiana State University, Baton Rouge, Louisiana, United States of America; College of Charleston, United States of America

## Abstract

Herbivory is a fundamental process determining reef resilience, and while algal farming damselfishes can help shape benthic assemblages, an understanding of their contribution to areas outside of defended territories is relatively unexplored. Here, we demonstrate how the farming damselfish *Stegastes marginatus* plays a dual role in benthic structuring by 1) contributing to persistence of the invasive macroalga *Acanthophora spicifera* within a Hawaiian marine protected area, where the macroalga occurred exclusively inside *Stegastes* territories, and 2) behaving as an opportunistic browser of the exotic alga outside their territorial borders. Greater than 50% of the biomass of tethered *A. spicifera* was consumed within one-hour when placed outside *Stegastes* territories, compared to <5% lost from tethers within territories or herbivore exclusion cages. *In situ* remote video revealed that tethered *A. spicifera* located outside territories was grazed primarily by the surgeonfish *Acanthurus nigrofuscus* (∼68% of total bites) and, surprisingly, by *S. marginatus* (∼27% of total bites) that left their territories to feed on this resource on 107 occasions during 400 min of filming. Further, for over half of those occurrences where *S. marginatus* grazed on the tethered macroalga outside of territories, they fed alongside conspecifics and other species, displaying little of the aggressiveness that characterizes this damselfish. These results show that *S. marginatus* plays a wider role in determining benthic assemblages than previously recognized, acting both as cultivators of a canopy-forming invasive macroalga within their territories, and as opportunistic browsers in neighboring sites. Consequently, *S. marginatus* can affect benthic species composition across their territory borders. These results provide a rare example of interspecific facilitation of an exotic alga by an indigenous marine fish. Accounting for fish behaviors more broadly is important to further our understanding of ecological processes that shape reef ecosystems to improve management of MPAs that often support extensive farming damselfish populations.

## Introduction

Herbivory by reef fishes profoundly influences the biomass, diversity, and canopy height of algal assemblages, typically resulting in coral-epilithic algae dominated communities [1], [2]. However, studies that examine how the range of behaviors exhibited by herbivorous fish species contributes to shaping coral reef ecosystems are limited [3]–[6]. Worldwide declines in coastal fish populations have had cascading effects on benthic reef communities, with the loss of herbivores credited as a key factor driving phase shifts on coral reefs towards assemblages overgrown with fleshy macroalgae [1], [2], [7]–[9]. Hawaiian coral reefs have been further exacerbated by herbivore losses because the fleshy macroalgae that typically replace corals are often exotic ( = invasive) species [10], [11]. Even highly palatable invasive species, such as the red alga *Acanthophora spicifera*, readily proliferate on Hawaiian coral reefs where herbivore abundance is reduced [12]. Thus, knowledge of how herbivore behaviors shape species composition is crucial for a more comprehensive understanding of reef ecosystem dynamics and implementation of management strategies to preserve them.

The identities of herbivores and their relative impacts in the Hawaiian Archipelago, as well as other reef systems, have primarily been inferred from field surveys, feeding assays and gut content analyses [12]. When herbivore behavior is considered, fishes are typically designated as exclusively farmers or foragers [3] with most foragers largely grazing on turf algae while fewer species consume macroalgae (i.e. browser) [13]. Research utilizing *in situ* remote video recordings, however, has captured novel foraging behaviors and revealed more nuanced relationships between coral reef algae and herbivores than previously understood [4], [14]–[16]. In one compelling example, *in situ* remote video clearly showed that a single species, regarded as an uncommon invertebrate consumer (dusky batfish *Platax pinnatus*), was in reality the primary consumer responsible for the reversal of an experimentally induced phase shift from fleshy macroalgae to coral-epilithic algae [4]. How the potential range of fish behaviors, including latent ones, may affect coral reef resilience is poised to receive further consideration.

While herbivorous fishes contribute to coral reef communities by continually cropping back algal canopies as foragers, one prevalent guild of herbivores, the territorial farming damselfishes (Pomacentridae), are an exception to this paradigm because these fish typically increase macroalgal standing biomass, productivity and diversity on coral reefs [17]–[21] by promoting growth of palatable macroalgae and defending it from other grazers [22]–[24]. As a result, territorial damselfishes have received considerable attention. Algal farming damselfishes typically maintain territories with distinct benthic assemblages by aggressively defending their cultivated resources from other herbivorous fishes, which has led to suggestions that farming damselfishes may function as keystone species [18], [25]. The visible contrast between benthic assemblages inside damselfish territories versus adjacent, undefended areas is often striking [23], [26]. However, behavior of farming damselfish in shaping reef assemblages outside their territories has rarely been considered. The two exceptions were observations that *Stegastes planifrons* does not feed outside its territory [19] and *Dischistodus prosopoteania* attempted to defend a palatable macroalga tethered outside of its territory [23]. Examining *in situ* behaviors of farming damselfish using remote video can further our understanding of ecological processes that shape coral reef ecosystems and improve management of coral reefs, including marine protected areas (MPA) that support extensive populations of farming damselfishes.

The goal of this study was to use *in situ* remote video recordings and benthic cover analyses to examine what role the algal farming damselfish, the Hawaiian Gregory *Stegastes marginatus* ( = Pacific Gregory *S. fasciolatus*), may have on the invasion success of the exotic macroalga *A. spicifera* in a Hawaiian MPA.

## Materials and Methods

### Ethics statement

The State of Hawaii Department of Land and Natural Resources (DLNR) issued Special Activity Permit No. 2010-51 for this field study because it took place in Pupukea Marine Life Conservation District (MLCD), property that is owned and administered by the DLNR.

### Study site and benthic characterizations

Observations and experiments were conducted in tide pools (<1.5 m depth) of the Pupukea Marine Life Conservation District, a MPA on the North Shore of Oahu, Hawaii (N21°38′5″ W158°3′48″) established in 1983. To characterize and compare benthic species composition inside versus outside *S. marginatus* territories, 15 haphazardly chosen *S. marginatus* territories and 15 nearby non-defended areas were mapped, labeled and photographed. *Stegastes marginatus* territories were identified by noting the extent and spatial orientation of each resident's farmed patch(s), defined as the substrate where the majority of maintenance takes place [27]. Using a camera mounted on a fixed distance photoquad stand [28], standardized 147 cm^2^ images (3–4 images per territory) of the substrate were taken haphazardly within farming patches. To characterize benthic cover outside territories, photographs (3–4 images per area) were taken adjacent (ca. 1–5 m away) to *S. marginatus* territories where other fish species were seen grazing without being chased by the damselfish, and were matched in terms of depth, hydrodynamic conditions, and benthic topography to the inside territory areas. Images were taken in June 2010 and then repeated in the same 15 paired sites in September 2010. Photographs were analyzed using Coral Point Count (CPCe V3.6) [29] by projecting 30 stratified random points per image, and identifying the taxa beneath each point as turf algae, *A. spicifera*, other macroalgae, coralline algae, other live (i.e. urchin, coral) or sand. Field notes taken concurrent with photoquad sampling were used to confirm identifications. Percent cover values from the multiple images taken within a single territory or non-territory areas were then averaged to yield a mean percent cover for that location. As the primary goal here was to test for differences in *A. spicifera* abundance between inside and outside the *S. marginatus* territories, and finding that *A. spicifera* was completely absent (percent cover = 0%) outside of all the territories (confirmed by multiple site visits in October 2009 and April to September 2010; searched for at least 30 min per visit), statistical tests on percent cover data were not necessary.

To estimate the biomass of *A. spicifera* (wet weight) that was maintained inside *S. marginatus* territories and to test whether it persisted through time, all of this macroalga within a randomly placed 10 cm^2^ quadrat was collected, blot dried and weighed to the nearest 0.01 g. Biomass samples were collected in April–June and again in September 2010 from 3–4 of the mapped territories with one quadrat per territory per sampling period. Also, the areal coverage of *A. spicifera* was measured in the 15 mapped (photoquad) territories using flexible tape measures that could be laid along the reef contours. To account for the irregular shapes of *A. spicifera* patches inside the damselfish territories, patch area was measured to the nearest 1 cm by positioning one weighted tape measure along the patch length as a reference and then used a second tape measure to record patch width at 10 cm intervals. Further, because our scientific collecting permit specified that only *A. spicifera* could be manipulated or sampled in the MPA, canopy height was used rather than biomass to test for relative differences in macroalgae abundance inside and outside territories. Mean canopy height was determined by measuring canopy height of benthic algae at five haphazardly selected points within each of the 15 mapped territories and each of the 15 nearby non-defended areas.

### Fish grazing intensity

To determine relative grazing intensity on the invasive macroalga 5–7 branches (7–12 cm tall) were collected inside *S. marginatus* territories, blotted, weighed and then woven into tethers constructed of 20 cm long twisted raffia fibers with a weight (ca. 225 g) tied to each end. Tethered *A. spicifera* was randomly assigned to one of three different treatments: (1) outside *S. marginatus* territories to test the grazing rate of herbivores on the alga without damselfish defenses (outside territory; *n* = 7); (2) inside *S. marginatus* territories (inside territory; *n* = 7); and (3) controls consisting of a 1.3 cm^2^ mesh plastic-coated wire cage measuring 15×9×10 cm (cage control; *n* = 6) placed outside territories to account for any biomass loss due to handling because *A. spicifera* fragments easily [30]. A single 20 cm tether was used for each deployment and was placed in unique locations among trials within each respective treatment group. With 15 mapped territories and a total of 14 uncaged replicates (7 inside, 7 outside), each territory was used only once and randomly assigned to a treatment, leaving one unused territory. For inside territory replicates, tethers were placed haphazardly within ca. 20 cm radius of the center of a feeding patch. Before placing tethers in outside territory treatments, nearby resident fish were observed to determine the borders of the territory on the day of the experiment and then a tether was haphazardly placed in a neutral area ca. 1–1.5 m beyond the territory border. Care was exercised to avoid deploying tethers near any *A. spicifera* donor territories. All tethers were deployed for one-hour and then remaining macroalga was blotted and reweighed to determine percent change in wet weight.

### Behavioral observations

To identify and quantify interspecific differences in foraging behavior on the tethered macroalga treatments described above, an underwater camera (MicroVideo, #MVC2120-WP) was used to film browser activities for both inside and outside territory treatments for one-hour. The underwater camera was fixed to a low profile stand and cabled to an external digital camcorder (Canon Optura 20) that was positioned out of the water at least 15 m from a tether. Replicates were conducted singly and in random order over several days between the hours of 0800 and 1200 (i.e., 3–4 sequential deployments per day). This timing and experimental setup was necessary because our permit within this MPA specified that our trials be performed only during morning hours to minimize interaction with the public. From the video footage, the herbivore species, number of individuals grazing and total number of bites on the tethered macroalga were quantified independently by two observers. The number of occasions when one or more browsers were simultaneously feeding on the tethered *A. spicifera* was also quantified. Simultaneous feeding occurrences were defined as occasions when one *S. marginatus* was consuming tethered algae at the same time another fish (*S. marginatus* or other species) was also present at the tether. One of our original predictions was that *S. marginatus* might try to defend tethered macroalga in neutral areas and attempt to expand their territory border; if for example, they perceived *A. spicifera* as a valuable resource. Thus, to examine this possibility and test whether *S. marginatus* behaved aggressively while feeding on the tethered *A. spicifera* placed outside of their territories, the number of times they chased another fish away from the tether was also quantified. Bite rate values and grazing occurrences obtained by the two observers were then averaged for each replicate (differences between the two observers ranged from 0% to 12%). All statistical comparisons were made with SigmaPlot 11.0 (Systat, Inc., San Jose, CA.), and names of statistical tests and corresponding values are reported in the results.

## Results

### *Acanthophora spicifera* inside and outside of *Stegastes* territories

**Figure 1:**
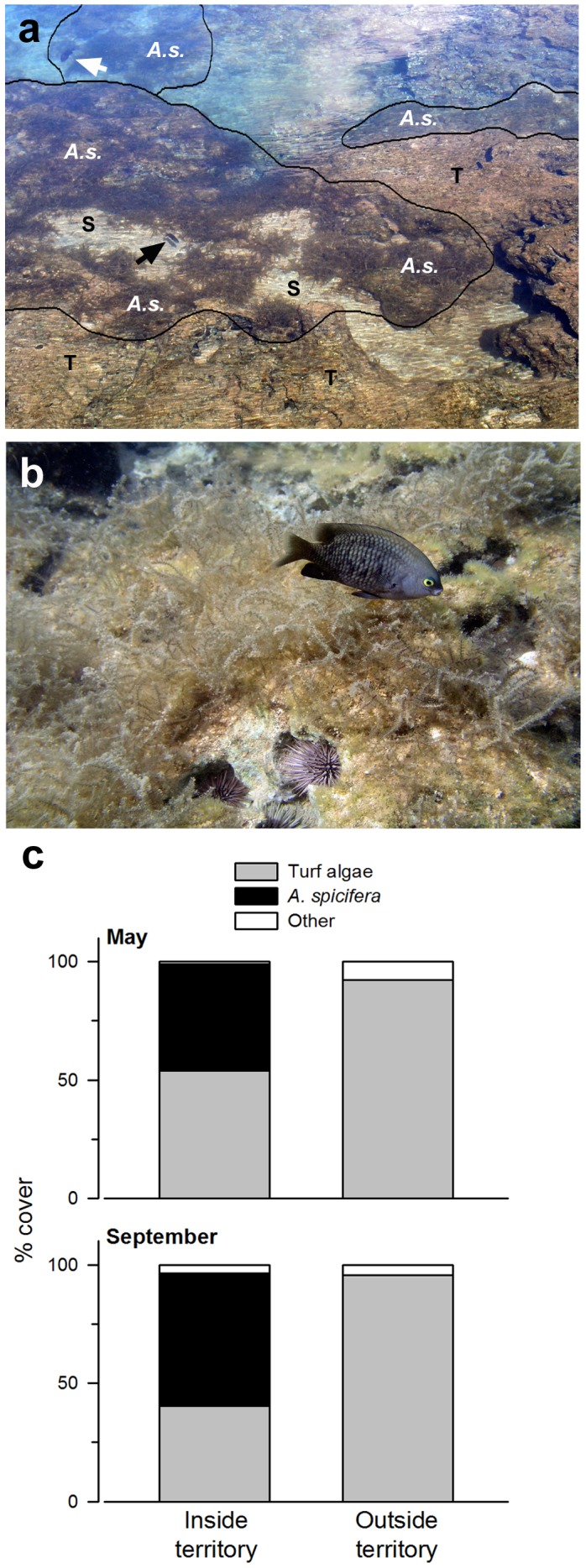
Invasive macroalga *Acanthophora spicifera* occurs exclusively within the territories of the algal farming damselfish *Stegastes marginatus*. A) Oblique view of *Stegastes marginatus* (arrow) feeding patches with dense growths of the invasive red macroalga *Acanthophora spicifera* (A.s.), turf algae (T) and sand (S). B) *S. marginatus* defending a lush canopy of *A. spicifera*. C) Percent cover of benthos inside and outside *S. marginatus* territories in May and September 2010.

Discreet patches of the invasive red alga *A. spicifera* occurred exclusively inside territories of the farming damselfish *S. marginatus* within the Pupukea MLCD (Fig. 1a & b). In May, feeding patches within the *S. marginatus* territories consisted of 45±4.3% (SE) *A. spicifera* and 53.9±4.3% filamentous turf algae (Fig. 1c). In contrast, outside territory areas were dominated by filamentous turf algae (92.1±2.3%), whereas *A. spicifera* (0%) was not distinguishable in photoquadrats or by close examination of the substratum *in situ*. Re-sampled in September, benthic composition remained different between inside (*A. spicifera*: 56.0±3.4%; filamentous turf algae: 40.3±3.3%) and outside (*A. spicifera*: 0%; filamentous turf algae: 92.2±2.1%) territory areas. Further distinguishing the two algal assemblages, the mean canopy height inside *S. marginatus* territories was 6.3±0.7 cm (SE) in April, 5.1±0.6 cm in May and 7.6±0.9 cm in September, whereas canopy height of algal assemblage was <0.5 cm outside territories across all sampling times.

**Figure 2:**
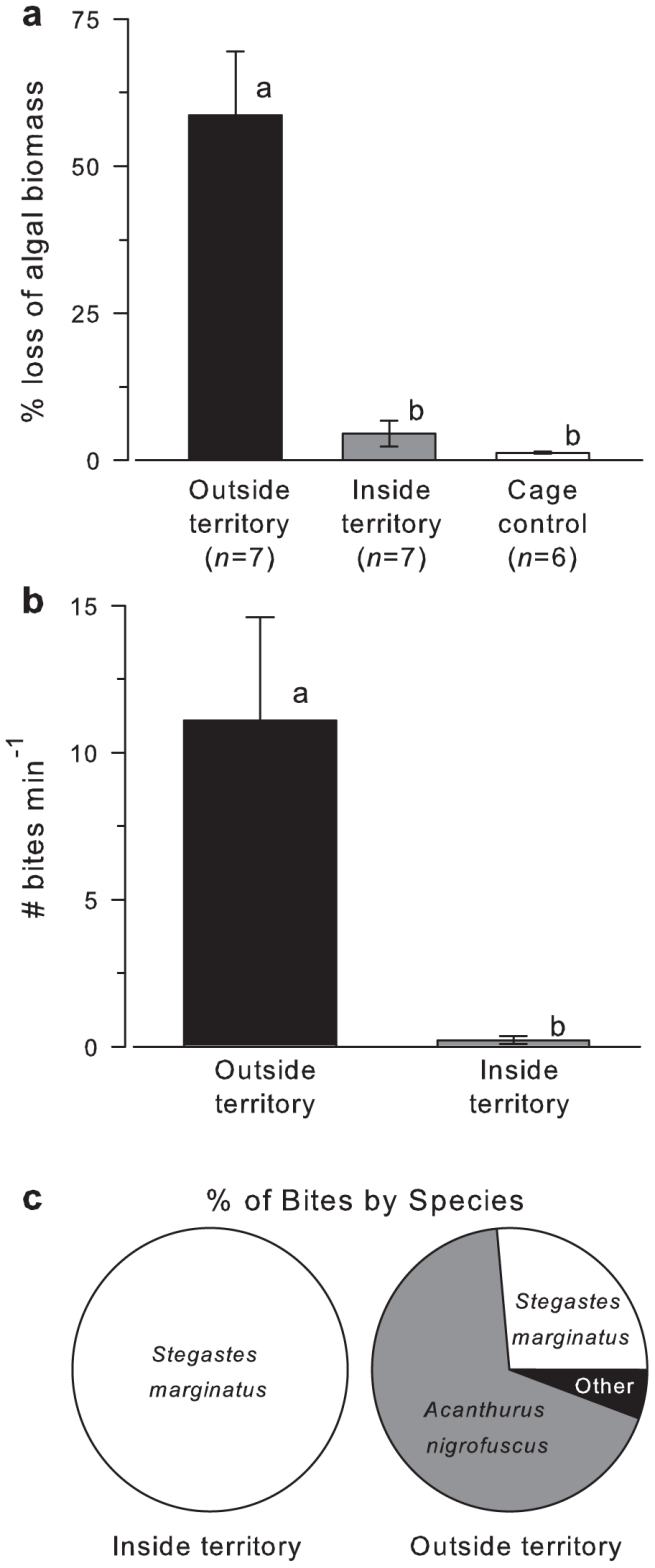
Herbivore grazing intensity on *A. spicifera* was highest outside the territorial defenses of *S. marginatus*. A) Percent loss of *A. spicifera* algal biomass from tethers placed inside *S. marginatus* territories, outside territories, or in caged controls. B) Number of bites min^−1^ on tethered *A. spicifera* placed inside and outside *S. marginatus* territories. C) Percentage of bites taken by different fish species inside and outside *S. marginatus* territories. Data are plotted as mean ± SE and bars with different letters indicate significant differences at *p*<0.05.

Average standing biomass of *A. spicifera* inside *S. marginatus* territories was 6.1±0.9 g wet weight 10 cm^−2^ (SE) in April; 11.5±2.3 g in May; 17.2±1.2 g in June and 8.2±1.7 g in September with tetrasporophytes (spore producing thalli) present throughout the study period. In the 15 territories measured in May, *A. spicifera* in the farmed patches covered 3,942.4±1800.1 cm^2^ territory^−1^ (range = 0–25,607.3 cm^2^ territory^−1^) with an average standing biomass of 2,168.5±99.1 g wet weight territory^−1^ (range = 0–14,084.1 g wet weight territory^−1^). Although wave exposure was not directly measured, the only damselfish territory without invasive seaweed was also the only territory located adjacent to the primary tidal channel and therefore subjected to higher water motion than the other 14 territories. Since the brittle thalli of *A. spicifera* easily fragment [30], the proximity of this territory to increased hydrodynamic action could account for the absence of the invasive alga in this area. Inside territories, *A. spicifera* was consumed at a rate of 4.5±2.2% h^−1^ (SE), while inside cage control biomass loss was negligible (1.3±0.2% h^−^1; Fig. 2a). When the invasive macroalga was placed outside territories, and removed from the defenses of *S. marginatus*, on average more than half of the biomass (58.7±10.8% h^−1^) was consumed within one hour and the percent loss was significantly higher than that of inside territories or cage controls (ANOVA, F_2,17_ = 23.63, *p*<0.001; Tukey's, *p*<0.001).

### Fish grazing inside and outside of *Stegastes* territories

To determine the fish species responsible for consuming the invasive macroalga and to compare their relative contributions, remote video recordings revealed that mean bite rates (bites min^−1^) were significantly greater when the tethered *A. spicifera* was placed outside (range = 6.1–31.9 bites min^−1^) compared to inside (range = 0–1.0 bites min^−1^) *S. marginatus* territories (t-test, t = −3.088, df = 12, *p* = 0.009; *n* = 7; Fig. 2b). Tethered *A. spicifera* within caged controls were not accessible to fishes and therefore not consumed. Inside territories, 100% of the few bites taken were by the resident *S. marginatus*, while outside territories, the majority of bites (67.9±7.1% SE) were taken by the surgeonfish *Acanthurus nigrofuscus* (Fig. 2c). Surprisingly, 26.5±7.6% of bites outside *S. marginatus* territories were also taken by *S. marginatus* that left their own nearby territories to feed on the undefended *A. spicifera* in adjacent areas. The remaining bites (5.6±4.3%) were taken by the Hawaiian sergeant fish (*Abudefduf abdominalis*), milletseed butterflyfish (*Chaetodon miliaris*), or convict tang (*Acanthurus triostegus*) (Fig. 2c).

**Figure 3:**
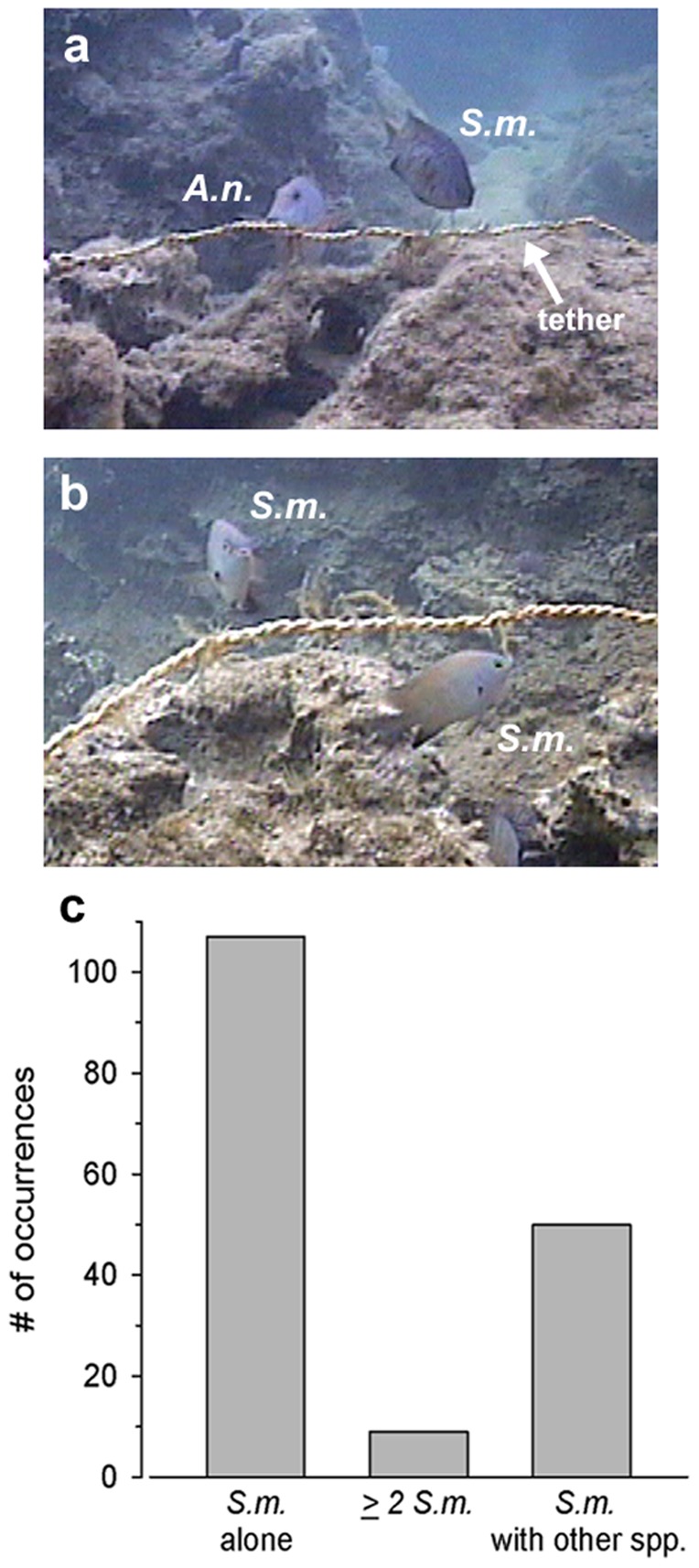
*Stegastes marginatus* acts as an opportunistic browser on *A. spicifera* outside its territorial borders. Examples of *Stegastes marginatus* feeding outside their territories on tethered *Acanthophora spicifera* alongside (A) the surgeonfish *Acanthurus nigrofuscus* (*A.n.*), and (B) another *S. marginatus* (*S.m.*). C) The total number of occurrences from 400 min of filming when *S. marginatus* was observed feeding on tethered *A. spicifera* either alone (*S.m.* alone), along with another *S. marginatus* (≥2 *S.m.*), or with another fish species in the outside territory treatments (*S.m.* with other spp.).

Remarkably, there were a total of 107 separate occasions when *S. marginatus* left their own nearby territories to feed on the tethered *A. spicifera* located in neutral areas (∼once every 4 min), a behavior that occurred in all 7 outside territory treatment locations by 2–5 different visiting fish per treatment location (Fig. 3). Total filming time for the outside territory treatments was 400 min, with 37.9±35.4% of that time spent by *S. marginatus* feeding on *A. spicifera* outside of their territories. Following deployment of the tethered *A. spicifera*, the average latency to first bite by a *S. marginatus* was 10.3±6.1 (SE) min. (range = <1–46 min), and by any herbivorous fish was 2.7±1.1 min. (range = <1–9 min). There were also numerous occasions (59 in 400 min) when *S. marginatus* fed on the tethered macroalga in the outside territory treatments along with either a conspecific (indicating that this treatment was consistently located in non-territorial areas) or another species (*A. nigrofuscus*, *A. abdominalis*, or *C. miliaris*) (Movie S1; Fig. 3). These simultaneous feeding events also occurred in all 7 outside territory treatment locations, and represented 23.0±26.7% of total filming minutes in these outside territory replicates. Because *S. marginatus* is compelled to continuously return to defend its own territory, it was also relevant to account for that time by partitioning out the total *S. marginatus* feeding minutes (e.g., all minutes with ≥1 *S. marginatus* feeding on *A. spicifera* in the outside territory treatment) to further discern the behavior of *S. marginatus* grazing in neutral areas. Surprisingly, 58.2±23.4% of total *S. marginatus* feeding minutes had one or more *S. marginatus* feeding with one or more other fish species. During simultaneous feeding events, aggression by *S. marginatus* was surprisingly low, as there were only 4 instances (6.8% of simultaneous feeding occurrences) when they chased another fish away from the tethered *A. spicifera*. This low aggression is in stark contrast to the typical defensive behavior of resident *S. marginatus* that vigorously chase away conspecifics or other herbivorous fishes such as *A. nigrofuscus* when they approach or enter their territory.

## Discussion

Using *in situ* remote video, our results demonstrate that an algal farming damselfish, *S. marginatus*, performs a dual function by shaping macroalgal assemblages both within and outside of its territorial borders, thereby serving as both a cultivator and an opportunistic browser, respectively. In contrast, earlier studies, which relied on other approaches, primarily focused on how algal farming damselfishes influence benthic composition *within* their guarded territories [18], [20], [22], [24], [25], [27] and rarely considered their ability to structure communities adjacent to their territories [19], [23]. Behaviors by these highly site-attached territorial fish, such as chasing intruding browsers, and weeding and fertilization, result in visually distinct and often more diverse algal assemblages within territorial borders compared to adjacent areas [17], [20]–[24], [27], [31]. *In situ* remote video is an effective approach used in herbivory experiments, revealing new insights into animal behaviors and plant-animal interactions on coral reefs [4], [15], [16], [32]. For example, a sleeping functional group was identified after a batfish displaying opportunistic herbivory was identified as the primary consumer responsible for reversal of a macroalgal dominated phase shift [4]. In our study, video recordings captured behaviors that showed *S. marginatus* can function in dual roles by shaping distinct benthic assemblages both as a farmer-defender of a palatable macroalga within its territory and as an opportunistic browser of the same algal species in neighboring sites. Without these video observations we would have incorrectly concluded that algal farming damselfish function only to harbor an introduced species inside their territories and thereby simply contribute to its invasion success. Consequently, our experiment demonstrates for the first time that a farming damselfish may influence algal assemblages outside its territories to a greater degree than previously recognized.

Our *in situ* remote video experiments also revealed a novel behavior of these territorial aggressive fish, namely, that *S. marginatus* shared available food resources with conspecifics and other herbivores when *A. spicifera* was located outside of their territorial borders. It is unlikely that this behavior is an attempt to expand the size of their territories because there was little evidence of *S. marginatus*-initiated aggression at the tethered *A. spicifera* placed in neutral areas. This is in stark contrast to the aggressive chasing typically performed by resident *S. marginatus* when other herbivores enter or swim nearby their territory borders [18], [25] [personal observations]. Although this farming species' aggressive behaviors during territory defense are well-documented, *S. marginatus* was not previously known to feed with other herbivores or exhibit resource sharing [18], [25], [33]. Despite the large number of studies on territorial farming damselfishes [24], there is only limited information on their behavior outside of their defended areas for comparison. For example, Hoey and Bellwood [23] noted that on a single occasion the farming damselfish *Dischistodus prosopotaenia* chased other herbivores grazing on the macroalga *Sargassum* that was experimentally tethered outside but adjacent to its territory, suggesting that it perceived it as a defendable resource rather than an opportunistic food source. Moreover, an Atlantic species, *Stegastes planifrons*, reportedly does not graze at all beyond its territorial borders [19]. Our experiment demonstrated that because *S. marginatus* grazed repeatedly alongside other fish species, as well as conspecifics, on a valued food resource when it was located outside the territory, that this damselfish species is an opportunistic browser, at least within the study site examined. In other taxa, foraging trips outside of a territory are often triggered by low food availability inside the territory [34], [35], however, our study provides a rare example of a territorial species traveling outside of its defended area to feed on an identical resource (*A. spicifera*) that is also abundant within the territory. Future studies are needed, however, to determine what consequences this has for the economics of territory defense in *S. marginatus*, and whether this phenomenon occurs in other farming damselfishes or in other reef locales.

Opportunistic browsing of *S. marginatus* occurs when a nearby resource is perceived as at least equivalent to the defended resources. In this example, the *A. spicifera* as a resource was clearly valued by *S. marginatus* because this macroalga proliferated inside of their feeding territories. Additional evidence of this macroalga's palatability is supported by the 107 occasions during the experiment that multiple *S. marginatus* individuals left their territories to graze on the rhodophyte in neutral areas. The strong feeding preference that *S. marginatus* displays for *A. spicifera* is also reflected by other non-fish herbivores in Hawaii [12], [36] including the green sea turtle [37], [38]. Damselfish territories function as refugia for macroalgae [17], [19]–[21], [23], [25] and results presented here are consistent with this conclusion because a conspicuous *A. spicifera* canopy was present only because it was protected from herbivory by *S. marginatus* (e.g., alga was absent outside of damselfish territories). To our knowledge, this is the first example of a farming damselfish specifically cultivating an exotic algal species. Algal farming damselfishes cultivate food species that occur nearby, however, these resources can be rare otherwise [21]–[23].

As an invasive species in the Hawaiian Islands, *A. spicifera* is a focus for management efforts [39]–[42] because its rapid growth can result in displacement of native species in coral reef habitats [43]–[45]. *Acanthophora spicifera* is also the most widely distributed invasive algae in the Hawaiian archipelago [45] even though many herbivorous fishes consume, and even prefer, this exotic over native species, such as *Padina japonica* and *Dictyospaeria cavernosa* [12]. The relationship between *S. marginatus* and *A. spicifera* is an interesting example of interspecific facilitation of an exotic species by a coral reef fish. Specifically, this facilitation is an example of mutualism because the interspecific interactions benefit both species [46]. As a highly palatable species [12], *A. spicifera* can only establish in sites, such as this Hawaiian MPA, where herbivore populations would otherwise exclude it because the macroalga has gained the tenacious defenses of *S. marginatus*. This is further supported by the fact that based on the intensity of grazing on *A. spicifera* tethered outside *S. marginatus* territories in the Pupukea MLCD, we estimate that 100% of the tethered *A. spicifera* would be consumed in approximately 1–6 hrs, a grazing rate that is comparable for this invasive species in other locations (e.g., Kaneohe Bay, Hawaii) [12].

Positive interactions also include reproductive mutualisms, in which one species benefits another, for example, by increasing propagule output [47]. By protecting the exotic rhodophyte from herbivory, *S. marginatus* enables *A. spicifera* to form canopies of adult thalli, which produce propagules, both as spores and vegetative fragments. Spore production in macroalgae contributes to both propagule dispersal as well as persistence in favorable areas [48]. This is important because after wave swells scour the brittle canopies of *A. spicifera* in Pupukea MLCD in winter, the macroalga has repeatedly regrown in the damselfish territories the following spring [personal observations, 2011–2014]. Further, the scoured canopies of *A. spicifera* become vegetative fragments that have the capacity to re-attach, also an important propagule dispersal strategy for this species [30]. Thus, the defenses of *S. marginatus* ensure the persistence of a reproductively-viable *A. spicifera* crop in this reef ecosystem.

As a case of a native species harboring an invasive one, the *S. marginatus* – *A. spicifera* relationship can inform management strategies, including within MPAs. Marine protected areas have been legislated to enhance fish stocks with the expectation that this action will increase browser densities and, in turn, curtail the expansion of fleshy macroalgae on coral reefs [49]–[51]. Here, we provide clear evidence that foraging fishes in a MPA serve as a biotic control for an invasive macroalga in Hawaii. On the one hand, this result is encouraging because, for example, a MPA on Maui (Kahekili Herbivore Fisheries Management Area (HFMA)) was established as a management tool to increase herbivore densities and thereby reduce the standing biomass and spread of fleshy macroalgae, including exotics such as *A. spicifera* (Department of Land and Natural Resources 2009). On the other hand, managers must contend with reefs that support both invasive macroalgae as well as colonial territories of *S. marginatus* because these fish territories can be a source of propagules of an exotic species within a marine managed area. Thus, the dual role of these farming damselfish as cultivators of invasive macroalgae within their territories and as opportunistic browsers of the same algal species in adjacent areas has broad implications for understanding the dynamics of reef ecosystems, invasion success of exotic macroalgae, and management.

## Supporting Information

Movie S1 **Video of the algal farming damselfish** ***Stegastes marginatus*** **(at right) feeding outside its territory on the tethered invasive macroalga** ***Acanthophora spicifera*** **alongside the surgeonfish** ***Acanthurus nigrofuscus*** **(at left).**
(MP4)
